# “Just put them in a group?” Teaching cooperation in childhood: Behavioural effects of a structured, tech‐supported intervention

**DOI:** 10.1111/bjep.70102

**Published:** 2026-06-05

**Authors:** Benedetta Zagni, Mark Van Ryzin, Dario Ianes, Sara Scrimin

**Affiliations:** ^1^ Department of Developmental Psychology and Socialisation University of Padua Padova PD Italy; ^2^ Centro Studi Erickson Trento TN Italy; ^3^ Oregon Research Institute Springfield USA; ^4^ Free University of Bozen Bolzano BZ Italy

**Keywords:** behavioural assessment, childhood, cooperation, tech‐supported cooperative learning

## Abstract

**Backgrounds:**

Cooperation is a core social skill essential for learning and citizenship, yet spontaneous cooperation in group settings cannot be taken for granted.

**Aims:**

This study investigates whether a structured, technology‐supported Cooperative Learning (CL) intervention can foster greater levels of observable cooperative behaviour in primary and middle school students.

**Sample:**

A total of 36 classes (containing 577 students ages 8–12) were randomly assigned to either an experimental or a control group.

**Methods:**

The intervention integrated key evidence‐based CL principles – positive interdependence, individual accountability, social skills training and group processing – into regular classroom instruction via a digital platform that supported teachers with automated group structures, role assignments and reflection prompts. Cooperation was measured behaviourally using a digital adaptation of the Marble Pull game, designed to assess real‐time, mutual cooperation in dyads.

**Results:**

Results from linear mixed‐effects models revealed a significant interaction between time and group: students in the experimental condition showed greater improvements in cooperative behaviour than those in the control group. Other interaction effects, and the main effect for age, were non‐significant.

**Conclusions:**

These findings provide behavioural support for the idea that cooperation can be explicitly taught and developed through intentional, structured pedagogy. They also highlight the importance of fidelity of implementation and teacher support. Overall, this study enhances our understanding of how digital tools and instructional design can promote prosocial development, providing practical implications for evidence‐informed educational practices.

## INTRODUCTION

Cooperation is a fundamental aspect of human social life and a core component of prosocial behaviour. It refers to the coordination of actions among individuals working toward a shared goal, in which mutual benefit is acknowledged and pursued (Bratman, 1992; Eisenberg & Miller, 1987). From a developmental standpoint, cooperative behaviour begins to emerge early in life, with signs of joint attention and shared intentionality observable in infancy, typically between 0 and 3 years of age (Warneken & Tomasello, 2007, 2009). However, the ability to understand the value of cooperation, to engage in it strategically and to sustain it across complex social interactions matures gradually throughout childhood (Grueneisen & Warneken, 2022). Especially during middle childhood, cooperation becomes more cognitively demanding and goal‐oriented, as children acquire a deeper understanding of others' perspectives, social norms and long‐term consequences of their actions (Gülseven et al., 2021; Laible et al., 2014). Overall, cooperation requires the ability to balance one's own needs with those of others (Gülseven et al., 2023). As such, it lies at the core of functioning in any social environment, including the classroom. In everyday classroom life, cooperation may take the form of students jointly solving a math problem, co‐constructing a poster for a history project or negotiating roles during a science experiment. Even routine activities, such as taking turns during discussions or organising materials for a group task, require children to align their behaviour with that of their peers, anticipate others' needs and contribute toward a collective outcome. However, cooperation is not an automatic behaviour, particularly in childhood. It involves a set of complex cognitive, emotional and social skills that do not emerge spontaneously in group settings. Therefore, it is unrealistic to expect children to cooperate effectively merely by placing them together or assigning them shared goals. While (some) children clearly possess the capacity to cooperate and may do so spontaneously in certain contexts, research shows that in classroom group‐work settings cooperative behaviour does not reliably emerge or sustain itself without intentional structuring and instructional support. Instead, cooperation must be taught, promoted organized and practised over time. Like any other developmental skill, it requires guided support, repeated opportunities for engagement and a structured environment in which cooperative behaviours are modelled and reinforced (Gillies, 2016; Van Ryzin & Roseth, 2019; Zagni et al., 2025).

### Fostering cooperation in schools: Why and how to do it

Classrooms are among the first structured environments where children are expected to collaborate toward shared goals, often with peers of varying backgrounds, abilities and perspectives. As such, they are fertile ground for developing the social–emotional skills required to navigate increasingly complex interpersonal dynamics. When cooperative norms are explicitly valued and structurally embedded in classroom practices, students are more likely to adopt collaborative approaches as their default mode of engagement (Buchs et al., 2017; Gillies, 2014). It is desirable for a teacher to promote cooperation in the classroom, given that cooperation enhances motivation and engagement by fostering a sense of belonging and shared purpose (Johnson & Johnson, 2009) and contributing to a more positive and inclusive learning environment. Furthermore, cooperative experiences in school also prepare students for participation in democratic societies, where collaboration, mutual respect and collective problem‐solving are essential (Battistich et al., 2004). Promoting cooperation in the classroom, therefore, not only benefits immediate academic and social outcomes but also lays the groundwork for active, prosocial citizenship.

However, a growing body of research has shown that the development of cooperative behaviours in group settings is influenced by contextual and instructional factors and can be supported through structured pedagogies such as Cooperative Learning (CL) (Gillies, 2016; Van Ryzin & Roseth, 2019). This approach aligns with the principles of evidence‐informed education, which emphasize the integration of the best available research evidence with professional expertise and contextual factors to inform instructional decisions (Dell'Anna et al., 2023; Vivanet, 2013). Within this framework, CL is not only a pedagogical choice but also an evidence‐based strategy that has been shown to effectively promote prosocial development and academic engagement when implemented with fidelity. Evidence from intervention studies consistently shows that students who participate in structured cooperative learning experiences are more likely to engage in prosocial behaviours and develop cooperation skills (Buchs et al., 2017; Johnson & Johnson, 2009; Van Ryzin et al., 2020). This is especially true when cooperative activities are designed with clear roles, mutual goals and mechanisms for feedback. Importantly, the success of these approaches hinges on the fidelity of implementation of CL: merely placing students in groups without structured interaction tends to reinforce individualism or passive participation (Gillies, 2004). Indeed, unstructured group work can lead to social loafing, dominance by a few members or even interpersonal conflict (Buchs et al., 2017; Gillies & Boyle, 2011).

### Key design principles for effective cooperative learning

Cooperation must be intentionally cultivated through evidence‐based design principles. Decades of research, most notably by Johnson and Johnson (2009), have identified core elements essential to effective CL: positive interdependence, individual accountability, explicit teaching of social skills and group processing.

These structural components are critical to transforming group work into a cooperative experience. For example, positive interdependence ensures that students perceive their success as linked to that of their peers, encouraging mutual support and joint responsibility. Individual accountability guarantees that each student remains engaged and contributes meaningfully to the group's task. Group processing helps students reflect on both their academic performance and the quality of their collaboration (Johnson & Johnson, 2013; Zagni & Van Ryzin, 2024). Explicit teaching of social skills supports students' development of key interpersonal competencies, such as active listening, turn‐taking or help seeking, since CL structures offer authentic, recurring opportunities to practise and refine these abilities within meaningful peer interactions (Zagni et al., 2025).

There is a great deal of research supporting the hypothesis that these design elements are critical to supporting the development of cooperative behaviours. For example, a study by Gillies (2004) found that students engaged in CL activities exhibited increased helping behaviours and more positive peer interactions compared to those in traditional learning settings. Similarly, Johnson and Johnson (2009) reported that CL not only improves academic achievement but also promotes interpersonal skills and mutual support among students. A meta‐analysis by Roseth et al. (2008) emphasized that the effects of CL are attenuated when fidelity of implementation is lower. These findings underscore the importance of implementing high‐fidelity CL strategies to cultivate cooperative behaviours in educational environments.

More recent studies continue to support these findings. For example, a study by Gillies (2016) found that CL strategies lead to improved student engagement and cooperation. Additionally, Van Ryzin and Roseth (2019) conducted a cluster‐randomized trial involving 15 middle schools and found that CL not only reduced bullying but also increased peer‐relatedness and empathy, suggesting that structured group activities can foster a more prosocial, inclusive and supportive classroom environment. Furthermore, research by Buchs et al. (2017) highlights that the success of CL depends on the fidelity of implementation, emphasising the need for clear roles, mutual goals and structured interactions. Their findings indicate that when CL is implemented with attention to these elements, students are more likely to engage in meaningful collaboration and develop essential social skills.

Although CL is widely supported by empirical research as a strategy to foster cooperation among students, its implementation in authentic classroom contexts often falls short of theoretical models. Teachers frequently encounter difficulties in managing group dynamics, ensuring balanced participation and maintaining adherence to the core principles of CL (Buchs et al., 2017; Gillies & Boyle, 2011). Without these foundational elements, cooperative behaviours may not emerge, and group work risks becoming either superficial or counterproductive (Abrami et al., 2004; Roseth et al., 2008).

To overcome these challenges and support the development of authentic cooperation, the platform PeerLearning.net was designed to assist teachers in structuring and delivering CL activities with high fidelity to key design principles. The platform automates key processes, including group formation, task and role distribution and the facilitation of reflection phases, ensuring that cooperative structures are consistently and effectively applied across different lesson structures (Low & Van Ryzin, 2024; Zagni & Van Ryzin, 2024). By embedding key design principles into each lesson, PeerLearning.net fosters the kind of mutual engagement necessary for genuine collaboration.

Crucially, the system provides scaffolding not only for students but also for teachers, who are guided through the lesson flow and supported in monitoring group interactions. This reduces the cognitive and logistical burden typically associated with leading cooperative activities, allowing teachers to focus on facilitating meaningful peer interaction. The platform also includes features for group goal setting and processing, helping students reflect on how well they collaborated and how they can improve, an essential component in the development of long‐term cooperative dispositions (Johnson & Johnson, 2013).

### The present study

Despite the strong theoretical and empirical foundation supporting CL, especially when implemented with fidelity, there is still a notable lack of research examining its behavioural impact on students' cooperative actions in real classroom settings. Most existing studies rely on teachers' ratings, self‐report measures or focus on broad social–emotional outcomes (e.g., peer‐relatedness, empathy, reduced conflict), leaving open the question of whether structured CL interventions directly foster observable, task‐based cooperation, particularly in middle childhood, a critical developmental period for social skill acquisition. Indeed, a previous study by Zagni et al. (2025), also conducted on the same age range, found that tech‐supported CL enhanced students' social–emotional skills; however, the outcomes were measured through self‐report questionnaires, which may be subject to bias or social desirability effects. Given the interactive and relational nature of cooperation, there is value in complementing subjective measures with objective behavioural indicators (see, for example, Denham, 2015). To the best of our knowledge, there is a lack of research specifically examining the impact of technology‐supported Cooperative Learning on observable cooperative behaviours, particularly among children in this developmental stage.

Thus, the present study aimed to investigate whether the use of PeerLearning.net can foster the development of cooperative behaviours in students, using a behavioural assessment rather than relying solely on self‐report data. Specifically, the present study evaluates whether a tech‐supported, fidelity‐focused CL intervention can promote measurable changes in children's cooperative behaviour using objective, game‐based assessments.

## MATERIALS AND METHODS

### Study design and procedure

The study was conducted across multiple schools (primary and middle^1^) in northeastern Italy. A total of 36 classes were randomly assigned to one of two conditions: 18 to the intervention group (Group 1) and 18 to the control group (Group 2). Schools were enrolled voluntarily. The control group received no intervention during the study period. Still, it was offered the opportunity to participate in the program during the following academic year, serving as a wait‐list control. Students in the control group continued with regular classroom instruction (business‐as‐usual) during the study period and did not receive any additional training or intervention related to cooperative learning.

The intervention began with two preparatory training sessions for teachers, held during the summer before the start of the school year. These sessions, facilitated by the lead researcher (a psychologist) and a trained research assistant, focused on cooperative learning, social and emotional learning and the use of the PeerLearning.net digital platform (see Figure 1 for an overview of the intervention timeline). Following the initial training, teachers received remote support through reading materials and instructional video content to help them prepare for classroom implementation.

**FIGURE 1:**
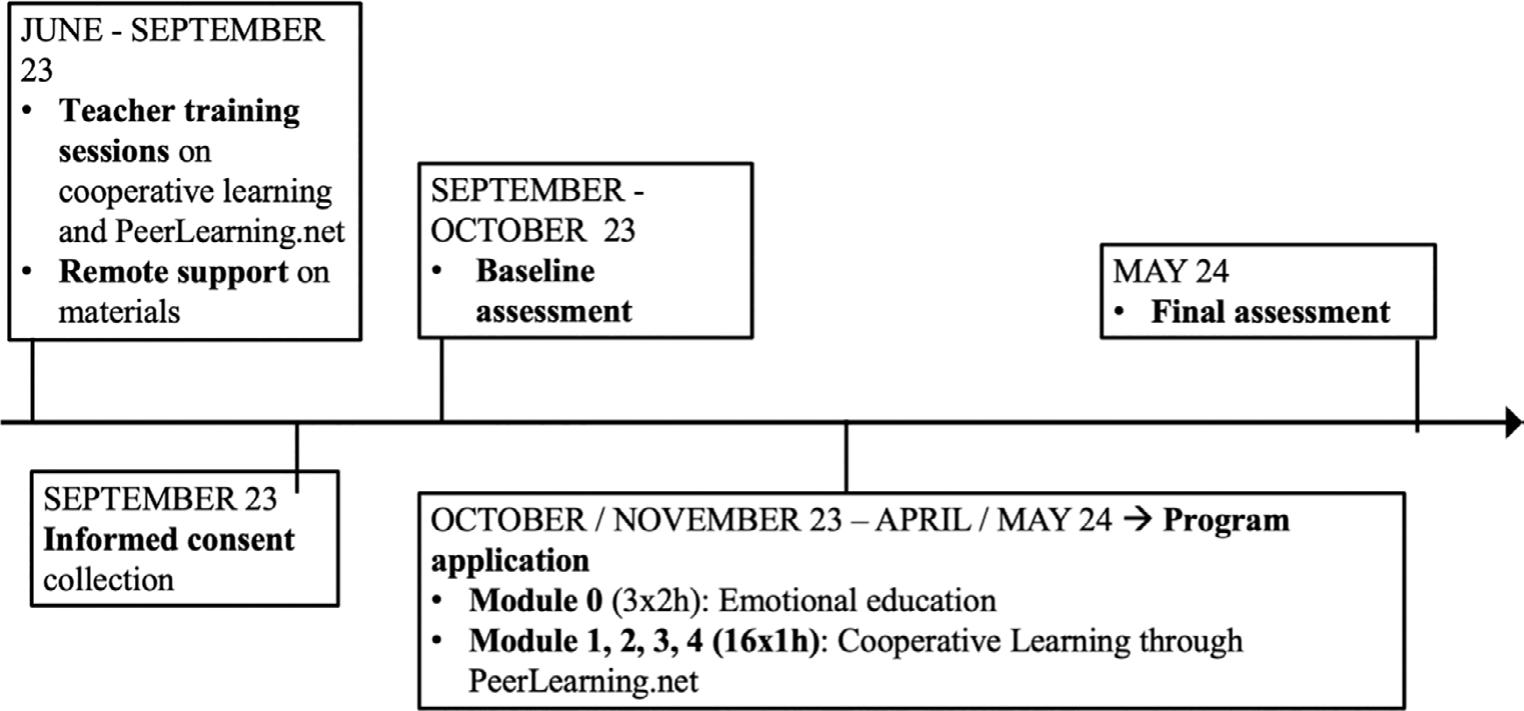
Timeline of the intervention.

Once the school year commenced, students in the intervention group participated in an initial set of four psychologist‐led classroom sessions (Module 0, October–November), designed to develop socio‐emotional competencies, including specific cooperative behaviours. These were followed by 16 cooperative learning lessons, integrated into regular reading comprehension instruction, delivered by Italian language teachers using PeerLearning.net software from November to May. During the first session, the research team supported teachers through job shadowing to ensure fidelity, after which teachers progressively assumed full responsibility for delivering the program. Support teachers^2^ were also involved, where available.

Data were collected at two time points (beginning and end of the school year) for both groups. The research protocol received approval from the relevant ethics committee (approval code: 96‐a), and written informed consent was obtained from parents. Pre‐ and post‐test assessments were administered to the control group using the same procedures, timing and measures as those used for the intervention group.

The administration of the cooperative game (see Measures section for details) took place in the classroom with all students present. The experimenter introduced the activity by informing the children that they would now play a partner‐based game, each using their own tablet. The goal of the game was described simply as trying to win as many marbles as possible, with a maximum of 10 marbles available across 10 trials. No explicit instruction regarding individual or dyadic goals was provided. A short practice phase allowed children to explore the task mechanics and to infer that coordinated action was required to maximize the number of marbles; practice trials were excluded from the analyses. No further instructions were provided to avoid priming the children toward cooperation or competition; this allowed them to independently discover that cooperation was the only effective strategy to obtain marbles. After the instructions, each student sat next to their assigned partner so they could interact freely during the game, including verbal exchanges if needed to coordinate their actions. This setup aimed to create a naturalistic context in which social behaviour could emerge spontaneously. Kids were allowed to talk during the game. At the end of the session, a brief debriefing was conducted to address any questions.

### Participants

A total of 607 students were initially recruited for the study, comprising 324 in the experimental condition and 283 in the control condition at Time 1 (pre‐test). By the time of the post‐test (Time 2), 314 students remained in the experimental group and 274 in the control group. To maintain data integrity and enable longitudinal comparison, only students who participated at both time points were retained for the final analyses. Indeed, students were excluded due to absence during one of the assessment sessions or school transfer during the academic year. Given the school‐based, year‐long nature of the project and the administration of a large battery of tasks, this conservative approach was adopted to ensure consistency across measures. The proportion of missing data was relatively small, and substantial efforts were made to recover absent students whenever possible. Multiple imputation was applied to all dependent variables with at least one missing value in the longitudinal dataset; five imputed datasets were generated (see Data Analysis). This resulted in a sample of 577 students: 307 in Group 1 (M_age_ = 9.48, SD = 0.79) and 270 in Group 2 (M_age_ = 9.63, SD = 0.79). The sample included an even distribution across educational levels, comprising eight fourth‐grade classes, eight fifth‐grade classes and two first‐year middle school classes in each group.

Specifically, the final sample comprised students from fourth‐grade (*n* = 223), fifth‐grade (*n* = 289) and first‐year middle‐school classrooms (*n* = 65). Mean age increased as expected across grade levels (fourth grade: *M* = 8.83, SD = 0.48; fifth grade: *M* = 9.82, SD = 0.45; middle school: *M* = 10.83, SD = 0.38). Classrooms varied in size, reflecting typical school composition. Within‐class age variability was generally low, as indicated by small standard deviations, consistent with age‐homogeneous classroom groupings.

### CL intervention and cooperative Behaviours

To foster cooperative behaviour, each lesson in the intervention was structured using one of three evidence‐based Cooperative Learning formats (group inquiry, group project and jigsaw), each of which incorporated all key design principles. These methodologies were selected for their effectiveness in promoting peer interaction, interdependence and collaborative engagement (Van Ryzin & Roseth, 2018). Regardless of the specific socio‐emotional skill targeted in each module – whether active listening, turn‐taking, help seeking/giving or conflict resolution – cooperation was a constant requirement embedded in the design of the learning environment.

All three CL structures emphasize positive interdependence and individual accountability, which are widely recognized as the foundational mechanisms of cooperation (Buchs et al., 2017; Johnson & Johnson, 2009). In group projects, students depended on one another to complete distinct but complementary components of a shared task (task‐based interdependence). In group inquiry, each child assumed a specific role that contributed to the group's problem‐solving process (role‐based interdependence). In the jigsaw format, each student was responsible for mastering and then teaching a portion of the content to their peers, reinforcing mutual reliance and equal participation. In all formats, the successful completion of the task depended on the active contribution of every group member.

This structure helped students understand, through direct experience, that achieving the lesson's objective required cooperation: not simply working side by side, but actively supporting one another to reach a shared goal. Rather than being an abstract ideal, cooperation became a necessity embedded in the task's logic: “*I can only succeed if you do too*.” This aligns with theoretical perspectives that define cooperation as coordinated action toward mutual benefit (Bratman, 1992; Gülseven et al., 2023).

Ultimately, teachers (supported by the researchers, in a job‐shadowing way) played a crucial role in fostering the development of these cooperative competencies. Within the framework of Vygotsky's Zone of Proximal Development (Vygotsky, 1978), they acted as active scaffolds, explicitly modelling cooperative behaviours, reinforcing them when observed and providing formative feedback when group dynamics faltered. Drawing on the principles of Positive Behaviour Support (Sugai & Horner, 2009), as promoted by PearLearning.net, teachers highlighted and rewarded verbal and nonverbal acts of cooperation, such as offering help, coordinating timing or facilitating group reflection. By making cooperation both visible and valued, and by reinforcing it systematically, the intervention allowed students to develop cooperative behaviours not just as incidental outcomes, but as explicit, teachable skills (Gillies, 2016).

### Measure

Cooperative behaviour: the Marble Pull Game.^3^ To assess cooperative behaviour, we implemented a digital version of the Marble Pull game (see Figure 2), initially developed by Madsen (1971) to examine cooperation and competition among children. In the traditional version of the task, two children are seated opposite each other at a table, with a marble placed in the centre. Each child holds a lever connected to a tray supporting the marble. To successfully retrieve the marble, both participants must coordinate their pulling action. If one child attempts to pull unilaterally or too quickly, the imbalance causes the marble to fall into a central hole, resulting in a loss for both players. Thus, the task inherently requires cooperation and turn‐taking for success (Madsen, 1971; Madsen & Shapira, 1970; see also Kagan & Madsen, 1971).

**FIGURE 2:**
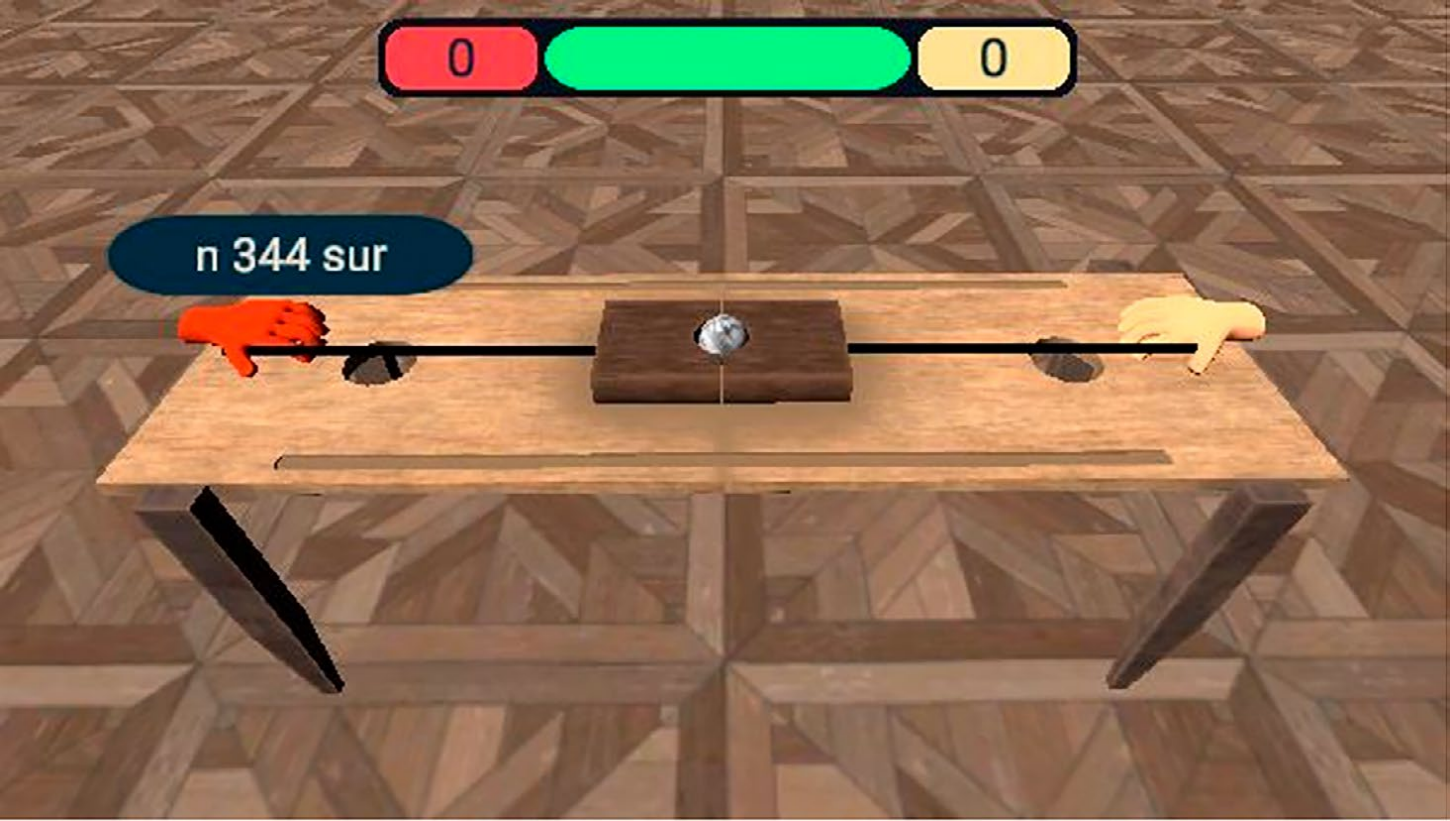
The marble pull game.

We translated this paradigm into a tablet‐based two‐player game developed using the Unity game engine (Unity Technologies, 2023). Each child interacted with the game individually on their tablet, connected wirelessly to ensure real‐time synchronisation. In the digital adaptation, a marble appeared between the players on the shared virtual interface. Both players had to press and hold a button on their screens simultaneously and for a sustained duration to retrieve the marble and allocate it successfully. If one player acted too soon, too late or released the button prematurely, the marble was lost. This mechanism preserved the original contingency of coordinated timing and cooperative intent. Before the main task began, a brief practice trial was conducted to ensure participants understood the mechanics of the game. During the experimental phase, each pair of participants had the opportunity to retrieve up to ten marbles. The number of marbles successfully collected was used as a quantitative index of cooperative behaviour, with a maximum possible score of 10 per dyad. Each child's score ranged from 0 to 10, reflecting the number of marbles successfully obtained. However, this raw score did not fully capture the quality of cooperation in each dyad. Specifically, it did not distinguish between perfectly balanced collaborations (e.g., 5–5) and highly asymmetric outcomes (e.g., 9–1), despite both resulting in the same total score.

To address this limitation, we computed a normalized cooperation index that integrated both the amount of shared success and the degree of balance between the two participants. The index prioritizes outcomes in which both players receive a substantial and similar number of marbles, while penalising unequal distributions. The computation is based on the lower of the two individual scores (min) and subtracts a scaled penalty determined by the absolute difference between the two scores. The formula used is:

$$\text{index}=\frac{\left(\min-\frac{\text{difference}}{2\cdot \left(5-\min\right)+1}\right)+1}{6}$$

This approach ensures that cooperative outcomes are evaluated on a continuous scale from 0 (no cooperation: 0–10 or 10–0) to 1 (perfect cooperation: 5–5), with intermediate outcomes (e.g., 3–3 or 4–5) mapped accordingly. The resulting index provides a more sensitive and theoretically grounded measure of cooperative quality, taking into account both mutual benefit and equity.

### Data analysis

All statistical analyses^4^ were conducted in R (Version 2025.05.0 + 496, Posit Team, 2025) using the packages lme4, mitml, broom and performance.

Prior to conducting inferential analyses, we examined the distribution of the cooperation index to assess its overall shape and potential differences across groups and time points. Histograms revealed a positively skewed distribution, with most dyads showing low to moderate levels of cooperation at baseline, and visible shifts in distribution across sessions and groups (see Figures 3 and 4).

**FIGURE 3:**
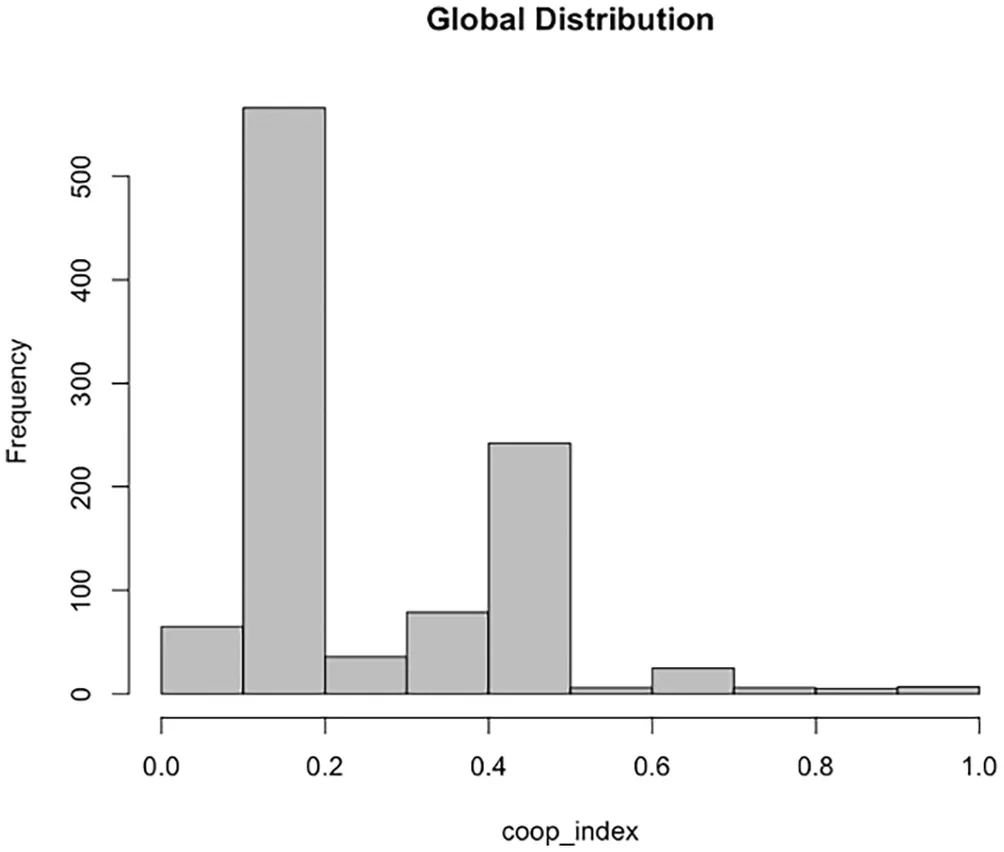
Global distribution of cooperation index.

**FIGURE 4:**
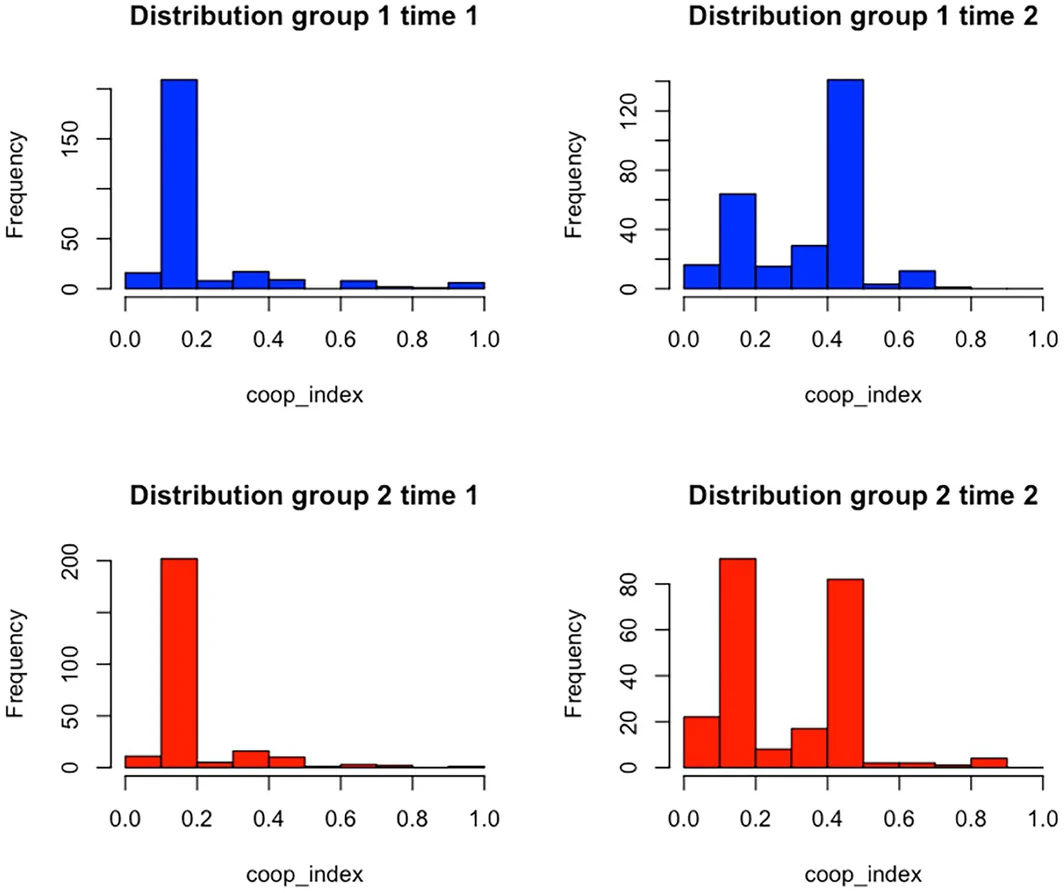
Group‐by‐time distribution of the cooperation index. Group 1 = intervention group; Group 2 = control group.

To investigate the effects of time, the experimental group and their interaction on cooperative behaviour, we conducted a linear mixed‐effects model on the normalized cooperation index. The model included fixed effects for time (Time 1 vs. Time 2), group (Group 1 vs. Group 2), their interaction and age (centred categorical covariate with levels 9, 10 and 11). Random intercepts were included for class and participant ID. The model was estimated using restricted maximum likelihood (REML) on multiply imputed data (5 datasets), and pooled estimates were computed using Rubin's rules (Rubin, 1987).

## RESULTS

In line with recommendations for mixed‐effects models, effect sizes are reported using marginal and conditional R^2^ values. The fixed effects results are reported in Table 1. A significant main effect of time was found, indicating higher cooperation at Time 2 compared to Time 1 (*β* = 0.178, *SE* = 0.015, *p* < .001). The main effect of group was not significant (*β* = −0.019, *SE* = 0.022, *p* = .384), nor were age effects. Crucially, the interaction between time and group was significant (*β* = −0.067, *SE* = 0.024, *p* = .006), indicating that the increase in cooperation over time was more minor in Group 2 compared to Group 1 (see Figure 5).

**TABLE 1:** Effects of time and group on cooperation index.

Predictor	Estimate	Std. error	*t*	df	*p*
Intercept	0.199	0.025	7.90	839.78	< .001***
Time (T2 vs. T1)	0.178	0.015	11.87	256.97	< .001***
Group (G2 vs. G1)	−0.019	0.022	−0.87	1103.33	.384
Age 9	0.0062	0.022	0.28	1033.62	.778
Age 10	0.0182	0.024	0.75	1058.10	.455
Age 11	0.0119	0.034	0.35	486.17	.723
Time × Group	−0.067	0.024	−2.81	66.99	.006 **

*Note*: *p* < .05 = **, *p* < .001 = ***. Group 1 = intervention group; Group 2 = control group.

**FIGURE 5:**
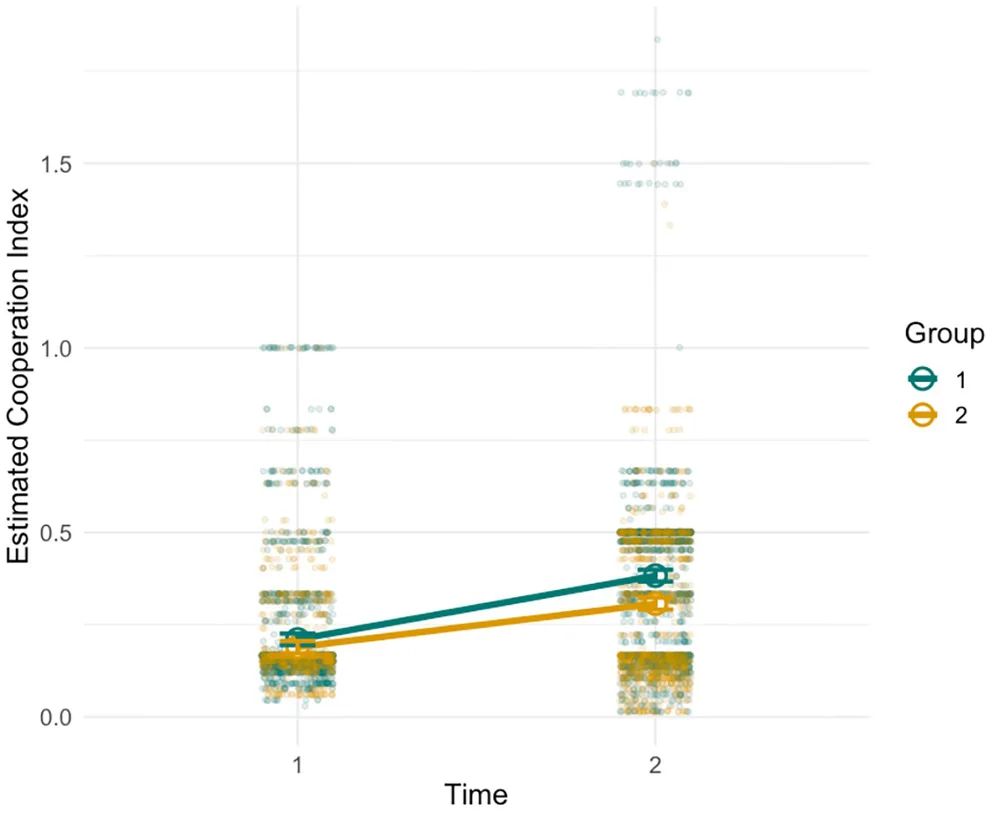
Interaction between time and group on cooperation index. Group 1: intervention group, Group 2: control group.

Model comparison using the D3 method (based on multiply imputed data) revealed that the full model explained significantly more variance than the null model, *F* (6, 875.21) = 27.58, *p* < .001. The pooled marginal *R* ^2^ (fixed effects only) was 0.153, and the conditional *R* ^2^ (fixed + random effects) was 0.247, indicating that both fixed and random effects contributed meaningfully to the explained variance.

To further unpack the interaction between time and group, we conducted follow‐up analyses to examine simple effects. Estimated marginal means revealed that both groups improved their cooperation scores from Time 1 to Time 2, but the magnitude of this improvement differed notably. In the intervention group (Group 1), cooperation increased from 0.210 to 0.383 (Δ = 0.173), whereas in the control group (Group 2), the increase was from 0.190 to 0.307 (Δ = 0.117).

Additionally, we compared the two groups at each time point. At baseline (Time 1), no significant difference was observed between the groups (*p* = .375), indicating similar starting levels of cooperation. However, at post‐test (Time 2), the difference between groups became significant (*p* < .001), with the intervention group showing substantially higher cooperation scores.

## DISCUSSION

The present study investigated whether a structured, technology‐supported Cooperative Learning intervention could foster observable cooperative behaviours among elementary and lower‐secondary students. Using a behavioural measure, the digital Marble Pull game, we assessed cooperation as an interactive and contingent skill, capturing not only the quantity but also the quality of mutual engagement. Our results showed a significant increase in cooperative behaviours over time, particularly in the intervention group, suggesting that structured CL, when implemented with fidelity, can foster the development of cooperative skills.

First, age did not moderate the intervention effect, suggesting that the structured CL approach was similarly effective across the 8‐ to 12‐year age range in this sample. Furthermore, it should be noted that cooperative behaviour increased across the school year, regardless of group assignment. This finding, in line with the existing literature, suggested a general developmental trajectory whereby students' ability to engage in mutual coordination improves with age and experience (Grueneisen & Warneken, 2022; Gülseven et al., 2021). In parallel, this may partially reflect a familiarity effect: students may have become more comfortable with the structure and contingencies of the Marble Pull game during the second session, leading to improved coordination independent of intervention exposure. In this context, the time effect provides a necessary baseline. If all students improved with repeated exposure, it becomes critical to examine whether the rate or quality of that improvement differs between conditions.

Thus, the interaction between time and group further clarified and explained the effects in a broader sense. Indeed, it indicated that students in the experimental condition, who engaged in structured cooperative learning through PeerLearning.net, showed greater improvement in cooperative behaviour compared to their peers in the control group. This finding provided behavioural evidence that cooperation is not a spontaneous by‐product of group work (which was surely practiced weekly in the control group classes, as it is usually in Italian primary schools), but rather a teachable social skill that can be systematically developed through intentional, structured teaching methods. Although the observed effect size is small to moderate, this magnitude is consistent with research on complex educational interventions conducted in naturalistic classroom settings, where multiple contextual and interpersonal factors jointly influence behavioural outcomes. On one hand, the intervention's success can be attributed to the consistent application of key evidence‐based principles, which are embedded directly into the structure of each lesson. PeerLearning.net supported fidelity to these principles by guiding teachers in designing and managing group work that demands mutual coordination and shared responsibility. These results reinforced prior theoretical claims (Gillies, 2016; Johnson & Johnson, 2009; Van Ryzin & Roseth, 2018, 2019) and offered support for the view that structured, high‐fidelity cooperative learning environments actively cultivate prosocial collaboration in primary and middle schools.

On the other hand, the intervention's effectiveness can also be attributed to the practical value of evidence‐informed instructional models, particularly when paired with targeted, situated teacher support. In this study, one such support was the inclusion of expert‐led job shadowing, in which an experienced educator worked alongside classroom teachers to model and co‐facilitate structured cooperative lessons. This approach likely contributed to maintaining high implementation fidelity, a critical but often neglected factor in real‐world educational interventions (Buchs et al., 2017). By observing, reflecting and gradually taking ownership of the CL routines, teachers were able to internalize not only the “what” but the “how” of cooperative learning practice.

Furthermore, one of the strengths of the study was that, unlike prior studies that rely mainly on self‐reports (e.g., Zagni et al., 2025), this study employed an objective behavioural measure, addressing concerns about social desirability bias and providing a more nuanced assessment of cooperative quality. This methodological approach contributes not only to the cooperative learning literature but also to the broader field of educational psychology, where objective behavioural assessments of peer interaction are still relatively rare. The behavioural measure adopted in the present study aligns closely with prior experimental approaches that conceptualize cooperation as coordinated, interdependent action rather than as a dispositional trait or self‐reported tendency. Similar to classic paradigms developed in developmental psychology and comparative research (Madsen, 1971; Warneken & Tomasello, 2007, 2009), the Marble Pull task operationalizes cooperation through shared contingencies, mutual dependence and real‐time coordination between partners.

More recent behavioural studies have adopted analogous assumptions, using interactive tasks to capture cooperation as enacted joint action under conditions of interdependence (e.g., Mastromatteo et al., 2024; Zagni et al., 2024). Across these approaches, cooperation is defined by the ability to align one's actions with those of another individual in pursuit of a shared outcome, consistent with theoretical accounts of joint action and shared intentionality (Bratman, 1992; Tomasello et al., 2005). From this perspective, although the specific task used in the present study differs in format, it relies on the same core theoretical principles and behavioural contingencies underpinning widely used behavioural measures of cooperation. The consistency of our findings with this body of research supports the interpretation of the Marble Pull task as a valid indicator of cooperative behaviour, capturing the enactment of cooperation in interaction rather than reported attitudes or perceived competencies. Moreover, by using a real‐time interactive task, this study avoided common pitfalls of self‐report or teacher‐report measures, such as limited introspective accuracy or teacher‐rated halo effects (Wentzel & Watkins, 2002). It also provided a dynamic, situated view of cooperation as it unfolds in interaction, aligning with recent calls for more ecologically valid assessments of social–emotional skills (Denham, 2015). Importantly, the behavioural task captures cooperation as enacted coordination rather than as self‐reported disposition. This may reflect a more internalized form of cooperative competence, which is educationally meaningful because it indicates students' ability to translate cooperative norms into action during real classroom interactions. The normalized cooperation index offered an additional methodological advancement, as it operationalizes not only success but also how success is achieved, with balanced, mutually contingent action resulting in a higher score. This allowed for a more precise capture of what distinguishes mere task completion from genuine cooperative engagement.

Moreover, our findings reinforced the idea that cooperation is not an automatic outcome of group work, but rather a teachable skill, consistent with prior research (Gillies, 2016; Van Ryzin & Roseth, 2019). The intervention emphasized key CL principles ‐ positive interdependence, individual accountability, social skill development and group processing (Johnson & Johnson, 2009) ‐ which likely contributed to the observed gains. By embedding cooperation into the task structure and roles, students experienced firsthand that collaboration was not optional but integral to success (Buchs et al., 2017). This supported the theoretical framing of cooperation as coordinated, mutually beneficial action (Bratman, 1992; Gülseven et al., 2023). In addition to that, the intervention was not delivered independently but was integrated into learning tasks and thus did not take away from instructional time.

Finally, consistent with Vygotsky's sociocultural theory and the notion of the Zone of Proximal Development (Vygotsky, 1978), the PeerLearning.net platform provided both teacher and student scaffolding. Teachers were guided in structuring CL lessons and reinforcing prosocial behaviour, while students practised cooperation within a clear framework of interdependence. This structured support likely minimized the common pitfalls of unstructured group work, such as social loafing, dominance and conflict (Gillies & Boyle, 2011) and instead created conditions for authentic and balanced collaboration.

## LIMITATIONS AND FUTURE DIRECTIONS

Although this study is the first to investigate, from a behavioural measurement, the increase in cooperation through CL, various limitations should be acknowledged. First, the study employed a single behavioural task, the Marble Pull game, as the primary outcome measure. While this task captures coordinated, real‐time collaboration and has firm theoretical grounding (Madsen, 1971), it focuses on a specific type of dyadic cooperation. Broader assessments, such as open‐ended group tasks or socially complex classroom interactions, would provide a more comprehensive view of cooperative competence.

Second, the study was geographically limited to schools in northeastern Italy. Although the sample was age‐diverse, contextual variables such as school climate, teacher training or cultural attitudes toward group work may have influenced results. Replication in different educational systems and cultural settings is needed to determine the generalizability of the findings.

Third, although the intervention spanned several months, we captured only short‐term changes. The long‐term developmental impact of structured CL remains an open question. Future longitudinal studies should assess whether cooperative behaviours persist, generalize to new contexts or translate into broader social–emotional competencies.

Moreover, while the inclusion of job shadowing with an expert educator likely enhanced fidelity and supported effective implementation, representing a key strength of the intervention, it also poses a limitation in terms of scalability, as such intensive, personalized support may not always be feasible or replicable in typical school settings. A key limitation of the present study is that the intervention comprised multiple interconnected components, including teacher training, expert‐supported implementation, initial student‐focused lessons and the regular use of a digital platform to structure cooperative learning. As a result, it is not possible to disentangle the specific contribution of each element. Rather than evaluating the isolated effect of the platform, the present findings should be interpreted as evidence for the effectiveness of an integrated, fidelity‐oriented cooperative learning intervention.

Another limitation is the absence of active control groups that either implemented a different cooperative learning system or used group work without adhering to key CL principles. Including such comparisons would have allowed us to disentangle whether the observed benefits stem specifically from the PeerLearning.net platform, from the fidelity to cooperative learning design elements or from structured group work more generally.

Finally, integrating multi‐informant data could strengthen future evaluations. Combining behavioural tasks with teacher ratings, peer reports or physiological markers of self‐regulation (e.g., cardiac vagal tone) would enable a richer, multidimensional understanding of how cooperation develops and how it is best supported. Moreover, future research should explicitly model dyadic data (e.g., using partner effects, which were not tested in this study) to better capture how individual and relational factors jointly shape cooperative behaviour.

## CONCLUSIONS

This study provides behavioural hints that structured, technology‐supported Cooperative Learning has the potential to shape cooperative behaviour in classroom settings. Our findings support the view that cooperation is not simply elicited through group work but cultivated through intentional design. PeerLearning.net offers a promising model for embedding cooperative structures into educational practice to develop cooperative skills. Future research will be essential to deepen our understanding of how to sustain and scale such interventions for long‐term impact on social development.

## AUTHOR CONTRIBUTIONS

**Benedetta Zagni:** Conceptualization; methodology; data curation; formal analysis; project administration; writing – review and editing; writing – original draft; investigation. **Mark Van Ryzin:** Conceptualization; supervision; validation; methodology; writing – review and editing; resources. **Dario Ianes:** Conceptualization; methodology; validation; supervision; writing – review and editing; resources. **Sara Scrimin:** Conceptualization; methodology; writing – review and editing; project administration; formal analysis; supervision; resources; data curation; validation; writing – original draft.

## FUNDING INFORMATION

The authors have nothing to report.

## CONFLICT OF INTEREST STATEMENT

The authors declare no conflicts of interest.

## ETHICS STATEMENT

Ethical approval for this study was obtained from the University of Padua Ethics Committee, protocol number 96‐a.

## CONSENT

In accordance with ethical guidelines, written informed consent was obtained from all participants' legal guardians, and assent was obtained from the students involved in the study.

## Data Availability

The data that support the findings of this study are available on request from the corresponding author. The data are not publicly available due to privacy or ethical restrictions.

## Footnotes

^1^
The Italian education system is structured as follows: primary school lasts 5 years and starts at age 6, followed by lower secondary school for 3 years (ages 11–14). Upper secondary school lasts 5 years (ages 14–19) and includes various tracks such as lyceums, technical institutes, and vocational schools. At the end of upper secondary education, students take the national exam to obtain their diploma and access higher education.

^2^
In the Italian school system, *support teachers* (*insegnanti di sostegno*) are assigned to classrooms where students with certified disabilities are enrolled. Their role is to promote inclusion by providing individualized support tailored to the specific educational and social–emotional needs of these students. Support teachers collaborate with subject teachers to adapt instruction and facilitate the full participation of all students in classroom activities.

^3^
A video of the game is available on: https://drive.google.com/file/d/1‐zGnqvctcNc7dlnJpYpx16ncY1a3F_C5/view?usp=sharing.

^4^
Data analysis was supported by dr. Francesco Pacenza (University of Trento).
